# SLC7A5 act as a potential leukemic transformation target gene in myelodysplastic syndrome

**DOI:** 10.18632/oncotarget.6512

**Published:** 2015-12-09

**Authors:** Yan Ma, Jing Song, Bobin Chen, Xiaoping Xu, Guowei Lin

**Affiliations:** ^1^ Department of Hematology, Huashan Hospital, Fudan University, Shanghai, 200040, China

**Keywords:** myelodysplastic syndrome, miRNA, SLC7A5 gene, SKM-1 cell line, siRNA

## Abstract

**Objective:**

Myelodysplastic syndromes (MDS) are a heterogenous group of clonal hematopoietic stem cell disorders characterized by increased risk of leukemic transformation. This study identifies microRNAs(miRNA) and miRNA targets that might represent leukemic transformation markers for MDS.

**Methods:**

Based on our previously established nested case-control study cohort of MDS patients, we chose paired patients to undergo Angilent 8 × 15K human miRNA microarrays. Target prediction analysis was administrated using targetscan 5.1 software. We further investigated the function of target gene in MDS cell line using siRNA method, including cell proliferation, cell apoptosis, cell cycle and electron microscope.

**Results:**

Finally we screened a subset of 7 miRNAs to be significantly differentially expressed between the case (at the end of follow up with leukemic transformation) and control group (at the end of follow up without leukemic transformation). Target prediction analysis revealed SLC7A5 was the common target gene of these 7 miRNAs. Further study on the function of SLC7A5 gene in SKM-1 cell line showed that downregulation of SLC7A5 inhibited SKM-1 cells proliferation, increased apoptosis and caused cell cycle arrest in the G0/G1 stage.

**Conclusion:**

Our data indicate that SLC7A5 gene may act as a potential leukemic transformation target gene in MDS.

## INTRODUCTION

Myelodysplastic syndrome (MDS) are a heterogenous group of clonal hematopoietic stem cell disorders characterized by ineffective and dysplastic hematopoiesis and increased risk of leukemic transformation [1–4]. Patients evolving into leukemia respond poorly to chemotherapy and die within a short time. Hence, it is very important to find detectable marker to predict the probability of leukemic transformation once MDS has been diagnosed. MiRNAs (miRNA) are small regulatory, non-coding RNAs. It is believed that miRNAs primarily affect the stability of mRNA and/or the initiation and progression of protein translation [5–8]. Even though the biological function of miRNA is yet to be fully understood, it has been shown that miRNAs play multiple roles in cancer genesis, development, invasion and metastasis [9–12]. Compared with mRNA, miRNAs are more stable and specific so as to be an ideal tumor marker. However, there are few studies on miRNAs involved in MDS transformation [13–15].

Based on our previous study, we established a nested case-control cohort of MDS patients [16, 17]. At the end of the follow-up, patients progressed into leukemia were classified as the case group, whereas patients without leukemic transformation were classified as the control group. In this study, we studied the miRNAs profiling of bone marrow monoclonal cells of MDS patients in the case and control groups. We finally screened a subset of 7 miRNAs to distinguish the case and control group remarkably. By predicting target gene of above seven miRNAs using targetscan 5.1 software, we found that SLC7A5 gene is the common target gene of above7 miRNAs. As SLC7A5 gene is one of the leukemic transformation associated gene resulted from our previous study [17], we further investigated the function of SLC7A5 gene in MDS cell line by downregulating the expression of SLC7A5 gene using siRNA method.

## RESULTS

### miRNA spectra of the MDS patients in the case and control groups

**Figure 1:**
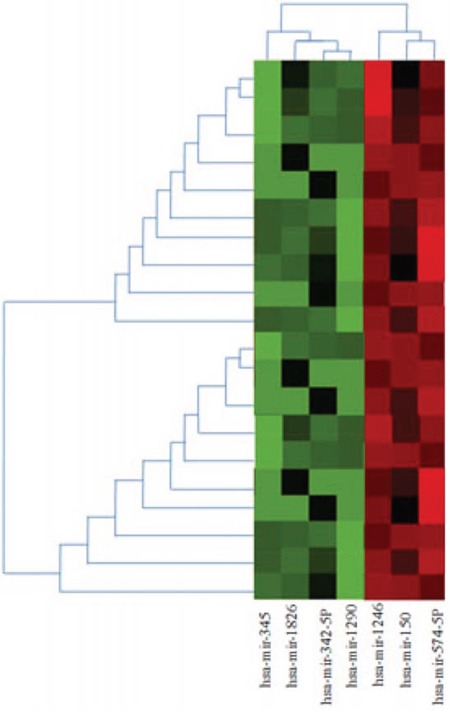
Heatmap and cluster analysis of miRNA expression in case and control group of MDS We selected miRNAs with *p* values of expressional differences less than 0.05 and/or >2-fold change between case and control group in miRNA array analysis. The heatmap generated by using heatmap software shows changes in the expression of these miRNAs in case group compared with controls. miRNAs whose expression is higher in case group are shown in red and those that are lower in green. The color key represents the values that are scaled to have a mean of zero and standard deviation of one. The identities of miRNAs are listed on the bottom, whereas the sample names are on the right.

From the above nested case-control study cohort of the MDS patients, paired patients were chosen for the gene expression microarray test (case = 10, control = 10) according to age, gender, WHO subtype, and IPSS cytogenetic subgroup, which are risk factors of leukemic progression in MDS patients (Table 1). We adapted a microarray platform from Agilent to profile the miRNA spectra. Excluding any miRNA with hybridization intensity <1.5 times the global mean intensity, there were20 miRNAs to be significantly differentially expressed between the case and control group, including 17 downregulated miRNA and 3 upregulated miRNA (Table 2). Cluster analysis of above 20 miRNAs resulted in a subset of 7 differentiall expressed miRNAs (Figure 1). By predicting target gene of above seven miRNAs using targetscan 5.1 software, we found that SLC7A5 gene is the common target genes of above 7 miRNAs. (Table 3), which was a MDS leukemic progression associated gene revealed in our previous study [17].

**Table 1:** The clinical characteristics of paired patient for miRNA microarray assay

**Group**	**WHO subtype**	**Gender**	**Age**	**Cytogenetcis**
case 1	RCMD	female	26	46,XX [20]
case 2	RCMD	male	48	46,XY [20]
case 3	RCMD	female	77	45,X,-X [16]/46,XX [4]
case 4	RCMD	male	49	46,XY [20]
case 5	RCMD	male	62	46,XY [20]
case 6	RAEB-1	male	70	46,XY [10]/46,XY,del(5)(q31q35),t(10;11)(p13;q23),add(18)(p11.2) [6]/47,idem,+19 [2]/46,idem,del(1)(p22),der(6)t(1;6)(p32;q22),der(10)t(10;11)add(10)(q24),+del(14)(q24) [2]
case 7	RAEB-1	female	73	46,XX [2]/46,XX,del(20)(q11.2q12) [70]/92,idemx2 [3]
case 8	RAEB-1	male	77	46,XY [5]
case 9	RAEB-2	female	56	46,XX [20]
case 10	RAEB-2	female	55	46,XY,del(20)(q11.2q12) [20]
control 1	RCMD	female	32	46,XX [20]
control 2	RCMD	male	42	46,XY,del(20)(q11.2q12) [20]
control 3	RCMD	female	76	46,XX,t(1;3)(p36.1;q21) [19]/46,XY [1]
control 4	RCMD	male	48	46,XY [20]
control 5	RCMD	male	69	46,XY [20]
control 6	RAEB-1	male	68	44,XY,der(5)t(5;11)(q13;q24),der(11)t(5;11)(?;q24),-18, add(20)(q11.2),-21,idic(22)(p11.2) [15]/88,idemx2 [4]/46,XY [1]
control 7	RAEB-1	female	70	44,XX,dic(2;4)(q34;p16),-6,add(11)(q23) [15]/46,XX [5]
control 8	RAEB-1	male	69	46,XY [6]
control 9	RAEB-2	female	50	46,XX [20]
control 10	RAEB-2	female	53	46,XY [20]

RCMD = Refractory Cytopenia with Multilineage Dysplasia; RAEB = Refractory Anemia with Excess of Blasts

**Table 2:** Detectable differential miRNAs in the case and control patient group by microarray

	**miRNA**	**Fold change between Case vs. control**	**Expression in case group**
1	hsa-mir-1290	0.26	down
2	hsa-mir-342-5p	0.22	down
3	hsa-mir-1224-5p	0.23	down
4	hsa-mir-345	0.38	down
5	hsa-mir-1228	0.38	down
6	hsa-mir-1249	0.32	down
7	hsa-mir-1826	0.26	down
8	hsa-miR-1306	0.38	down
9	hsa-miR-188-5p	0.43	down
10	hsa-miR-320a	0.48	down
11	hsa-miR-320c	0.26	down
12	hsa-miR-365	0.31	down
13	hsa-miR-423-5p	0.35	down
14	hsa-miR-483-5p	0.25	down
15	hsa-miR-634	0.31	down
16	hsa-miR-671-5p	0.23	down
17	hsa-miR-939	0.24	down
18	hsa-miR-1246	2.22	up
19	hsa-miR-150	10.41	up
20	hsa-miR-574-5p	8.04	up

**Table 3:** Prediction of target gene of 7 miRNAs screened by miRNAmicroassay

**MiRNAs**	**Target Gene Symbol**
hsa-miR-345	PUM2, PPP2R3A, BCAT1, ZFHX4, CHSY3, ARNT, SHE, **SLC7A5,** SOS1, ......
hsa-miR-1826	SP1, CSNK1G1, AMD1, SLC37A2, **SLC7A5,** PKD1, WNT4, XPO1, ELFN2, C16orf72, ......
hsa-miR-342-5p	AFF4, GSG1L, CACNB1, NPTXR, C1QTNF3, ITGA10, ATXN7L3, **SLC7A5**, ......
hsa-miR-1290	FOXN2, RSPO3, ZFP91, SLC39A9, PHF3, SLC12A6, STX6, TNFSF4, **SLC7A5**, ......
hsa-miR-1246	DMWD, C20orf27, FOXP4, CDC42SE1, BTBD14B, TMEM41A, GLP1R, SRM, KLHL3, CCDC64, ENC1, **SLC7A5**, ......
hsa-miR-150	HMP19, TBL1X, SRP9, PPARGC1A, ESRRG, LDR1, ZNF791, **SLC7A5**, ......
hsa-miR-574-5p	FOXP2, UBE2Q1, VGLL4, ARGLU1, YME1L1, CSDA, KCNMA1, **SLC7A5**, ......

### Down regulation of SLC7A5 inhibits proliferation of SKM-1 cell line

**Figure 2:**
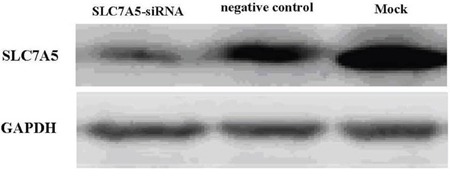
Western blot analysis of SLC7A5 protein The protein expression was further confirmed by Western blot analysis after three days of transfection. The levels of the SLC7A5 protein were significantly lower in SLC7A5-siRNA group than in the negative control group and Mock. In comparison, GAPDH protein did not vary markedly among the three groups.

**Figure 3:**
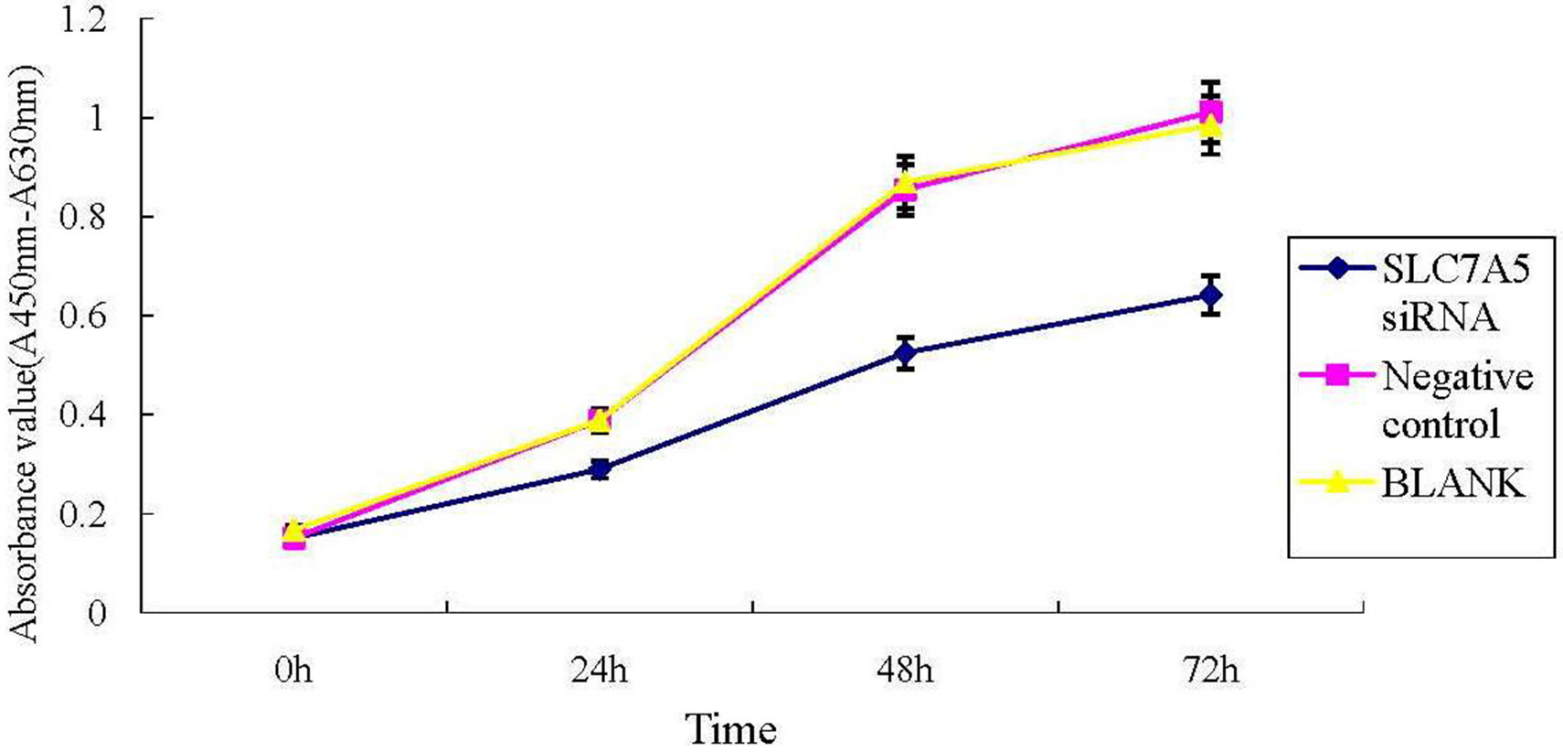
Down-regulation of SLC7A5 inhibits SKM-1 cells growth Cell proliferation of SKM-1 cells using CCK-8 assays. The growth of SKM-1 cells in SLC7A5-siRNA group was significantly inhibited compared with the negative control (NC) group (*P*<0.05).

Image Master Total Lab Image system analysis showed SLC7A5 expression in SLC7A5-siRNAgroup, negative control and blank group was 1662.63 ± 13.00, 4529.63 ± 24.36, and 6653.03 ± 18.76 respectively, indicating the success of down-regulation of SLC7A5 by SLC7A5-siRNA (Figure 2). To further study the function of SLC7A5 gene, SKM-1 cell line was investigated by down-regulating the expression of SLC7A5 gene using siRNA method. Cell proliferation rates were measured by CCK-8 assay. The growth of cells inSLC7A5 down-regulation group by SLC7A5 specific siRNA was significantly slower than that of the negative control group (Figure 3), demonstrating that down-regulation of SLC7A5 expression led to inhibition of growth of SKM-1 cells.

### Down regulation of SLC7A5 increases apoptosis of SKM-1 cell line

**Figure 4:**
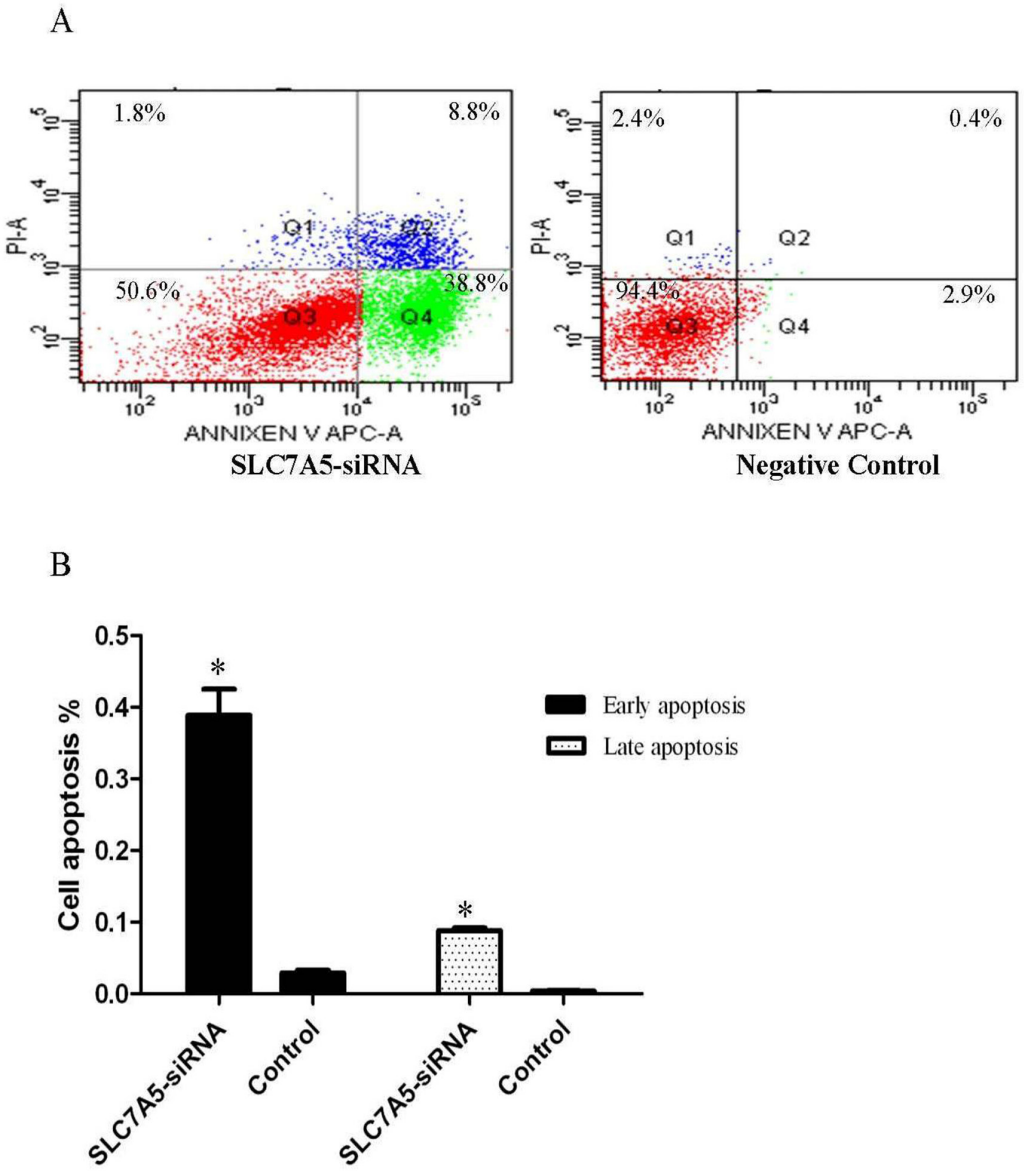
Down-regulation of SLC7A5 induces SKM-1 cells apoptosis Cell apoptosis analysis of SLC7A5-siRNA group and negative control group cells was analyzed by flow cytometry. **A.** Representative histograms of annexin V/PI double-staining flow cytometry. **B.** Percent apoptosis (including early and late apoptotic cells) determined by flow cytometry (n = 3). *P<0.05 vs control.

Cell apoptosis was assayed by flow cytometry. The percentages of early apoptosis cells in SLC7A5-siRNA group increased significantly compared with negative control group (38.9% ± 3.6%Vs 2.9% ± 0.4%). Also, the percentages oflater apoptosis cells in SLC7A5-siRNA group increased significantly (8.8% ± 0.4%Vs 0.4% ± 0.06%) compared with negative control group (Figure 4). Those outcomes revealed that downregulation of SLC7A5 promoted apoptosis.

### Down regulation of SLC7A5 caused cell cycle arrest in the G0/G1stage

**Figure 5:**
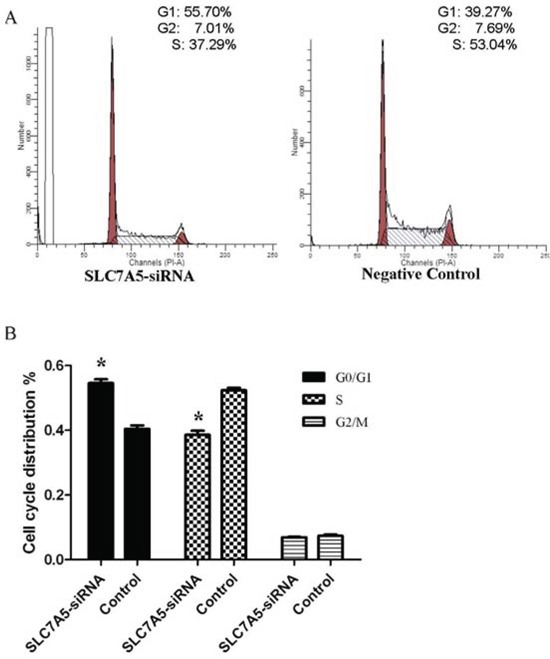
Down-regulation of SLC7A5 induces cell cycle arrest at G2/M phase in SKM-1 cells Cell cycle distribution of SLC7A5-siRNA group and negative control group cells was analyzed by flow cytometry with PI staining after 48 h treatment. **A.** Representative flow cytometry histograms. **B.** Cell distribution at G0/G1, S, and G2/M phases of the cell cycle (n = 3). *P<0.05 vs control.

The cell cycle analysis by flow cytometry illustrated SLC7A5-siRNA group cells in G0/G1 phase increased by 14% and cells in S phase reduced by 13% compared with negative control group cells (Table 4), (Figure 5) suggesting that cells of SLC7A5-siRNA group were obstructed at G0/G1 phase.

**Table 4:** Cell cycle assay by flow cytometry in SLC7A5-siRNAand negative control group

	**G1 phase (%)**	**S phase (%)**	**G2 phase (%)**
SLC7A5-siRNA	54.55 ± 1.16	38.55 ± 1.27	6.90 ± 0.13
negative control	40.36 ± 1.11	52.33 ± 0.73	7.31 ± 0.42
P value	0.01	0.002	0.18

### Electron microscopy

**Figure 6:**
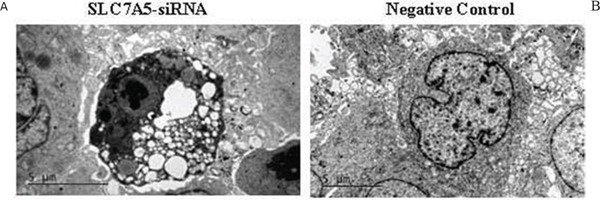
Down-regulation of SLC7A5 induces apoptosis in SKM-1 cells Ultrastructural changes in SKM-1 cells. **A.** SKM-1 cells without transfection. **B.** SKM-1 cells with TUBB-RNAi transfection. (magnification 94,000). There are plenty of lipid droplet (arrow) and mitochondrial swelling in SKM-1 cells with TUBB-RNAi transfection.

We also observed the ultrastructural changes in response to the down-regulation of SLC7A5in SKM-1 cells using electron microscopy. The SKM-1 cells of the control group were rich in mitochondria, which were normal in shape and size and had few lipid droplets (Figure 6A). In contrast, many mitochondria from SKM-1 cells with transfection were enlarged and showed morphological changes, including a less dense matrix, a large lipid droplet, mitochondrial swelling, condensed cytoplasm, and karyopyknosis (Figure 6B).

## DISCUSSION

To date, the prediction of the leukemic evolution of MDS is very difficult. Hence, a set of molecular markers enabling better prediction of disease progression and prognosis would be valuable. MiRNAs are small regulatory, noncoding RNAs and play multiple roles in cancer genesis, development, invasion and metastasis. Compared with mRNA, miRNAs are more stable and specific so as to be an ideal tumor marker. However, there are few studies on miRNAs involved in MDS transformation. Pons et al. detected miRNA expression in 25 MDS patients (high risk group = 3, low risk group = 22) and found that miR-155, miR-181a and miR-222 were upregulated in high risk patients [13]. Hussein et al. studied miRNA expression of 24 MDS patients (high-risk group = 6, low risk group = 18) and found that MDS patients with different cytogenetic abnormalities had different miRNA expression profiles. And they showed miR-221 (not miR-222) was downregulated (not upregulated) in high-risk MDS patients [14]. Choi et al. investigated the involvement of miRNAs encoded by chromosomes 8 and 1q in 65 MDS patients. They found a significant upregulation of miR-194-5p and miR-320a in MDS patients compared with controls. They further found that miR-194-5p is a candidate diagnostic biomarker for MDS and that low miR-194-5p expression could be associated with poor overall survival for MDS patients [15]. In order to avoid the selection bias and improve the efficiency of miRNA microarray assay, our study carefully chose case and controls from our previously established nested case-control study cohort for miRNA microarray analysis according to the risk factors identified from the multivariate analysis so as to improve the efficiency of miRNA microarray. We identified 20 miRNAs to be significantly differentially expressed between the case and control group, including miR-320a which is coincident with Choi et al. Of above 20 miRNAs, there were 17 downregulated miRNA and 3 upregulated miRNA, which was coincident with our previous study showing more upregulated genes than downregulated genes in case group [17]. We finally screened a subset of 7 miRNAs to distinguish the case and control group remarkably, including 4 down-regulated miRNA and 3 up-regulated miRNA. By predicting target gene of above seven miRNAs using targetscan 5.1 software, we found that SLC7A5 gene is the common target genes of above 7 miRNAs.

SLC7A5 or LAT1 (L-type amino-acid transporter 1) encodes amino acid transporters and is involved in the transportation of essential amino acids. The action of SLC7A5 correlates with cell growth. A previous study revealed the upregulation of SLC7A5 in many human primary tumors possibly because of the constant, high demand for nutrients in tumor cells. This result indicates that SLC7A5 may be a potential target for tumor treatment by downregulating the SLC7A5 expression to inhibit the proliferation of tumor cells [18–22]. Imai et al. reported the SLC7A5 overexpression in patients with stage I non-small-cell lung cancer (NSCLC), and the multivariate analysis showed that SLC7A5 expression is a promising pathological factor for the prognosis of patients with NSCLC [23]. Our previous study identified a subset of six genes associated with the evolution of MDS to acute leukemia, including SLC7A5 [17]. In order to further investigate the probable function of SLC7A5 in MDS, our present study on SLC7A5 gene in SKM-1 cell line showed that downregulation of SLC7A5 inhibited SKM-1 cells proliferation, increased apoptosis and caused cell cycle arrest in the G0/G1 stage, indicating that upregulation of SLC7A5 in MDS may increase the proliferation of malignant clones then accelerate the leukemic evolution.

In all, our study on MDS patients and MDS cell line strongly suggested that SLC7A5 gene may act as a potential leukemic transformation target gene in MDS, which will facilitate the prediction of MDS leukemic progression and may have clinical utility for the advanced identification of MDS patients with a propensity toward leukemia.

## MATERIALS AND METHODS

### Patients and ethics statement

Based on our nested case-control study cohort of MDS patients, we chose paired patients in 1:1 ratio according to risk factors for leukemic evolution. Case group included MDS patientswith leukemic transformation at the end of follow up and control group included MDS patients without leukemic transformation at the end of follow up. All patients gave their written informed consent. This study is in accordance with the modified Helsinki Declaration, and the protocols received approval from the ethics committees of the participating institutions (Institutional Review Board of Huashan Hospital, Fudan university).

### RNA extraction

The total RNA from bone marrow mononuclear cells was extracted using TRIzol (Invitrogen, Paisley, U.K.) according to the manufacturer's protocol. Aliquots of the RNA samples were conserved for quality evaluation using Agilent 2100 Bioanalyzer (Agilent Technologies, Palo Alto, CA).

### Array hybridization, data analyses and target prediction of miRNAs

To assess the level and composition of miRNA, miRNA arrays from Agilent Technologies were used. The RNA samples were labeled and processed according to manufacturer's recommended protocols. In brief, ≈100 ng of total RNA was dephosphorylated with calf intestinal alkaline phosphatase, followed by denaturing with heat in the presence of dimethyl sulfoxide (DMSO). A cyanine dye, cyanine3-cytidine bisphosphate (pCp), was then joined to the dephosphorylated single-stranded RNA (including miRNA) by T4RNA ligase. MicroBioSpin 6 columns (Bio-Rad) were used to remove any unincorporated cyanine dye from the samples. The purified labeled miRNA probes were hybridized to 8 × 15 K human miRNA microarrays in a rotating hybridization oven at 20rpm for 20 h at 55°C. After hybridization, the arrays were washed in Agilent gene expression (GE) Wash Buffer 1 with Triton X-102, followed by Agilent GE Wash Buffer 2 with Triton X-102. After washing, all slides were immediately scanned at 5 μm resolution by using a PerkinElmer Scan Array Express array scanner. The resulting images were quantified by using Agilent's Feature Extraction software. The differentially expressed miRNAs were identified by using Agilent miRNA gene arrays protocol. To increase the reliability of the data, miRNA species with hybridization intensities <1.5 times the average hybridization intensity (mean) were excluded from analysis. The miRNA clustering analysis was performed with the Hierarcical clustering algorithm provided in Multi Experiment Viewer, MeV4.0 software package (www.tm4.org/mev.html). After screening the differentiated miRNA, we predicted the targeting genes using targetscan software. Sites with P_CT_ > 0.75 are with higher probability of preferential conservation.

### SKM-1 cell line

Our lab purchased SKM-1 cell line from Health Science Research Resources Bank in Japan, and cultured in Roswel Park Memorial 1640 medium (Gibico Inc.) supplemented with 10% fetal bovine serum (Gibico Inc.) in a 37°C, 5% CO_2_ incubator.

### siRNA transfection

SKM-1 cells in log phase were divided into three groups. One group of cells was planted at a concentration of 5 × 10^5^ cells/well in 96-well plates and transfected with SLC7A5-RNAi (SLC7A5 downregulation group) in serum free medium. Polybrene was added to improve transfection efficiency as enhancing reagent. After 8 hours, the medium was changed for complete medium. The second group of cells was transfected with negative control (negative control group) in the same way as described previously. The third group of cells without intervention served as blank control group. After 3 days of transfection, transfection efficiency was examined using fluorescence microscopy and flow cytometry. Cells were harvested for further experiments.

### Western blot analysis

Approximately 2 × 106 cells were lysed in RIPA buffer (50 mM Tris-HCl, pH 8.0; 1 mM EDTA, pH 8.0; 5 mMDTT; and 2 % SDS) and total protein was extracted and determined with a BCA assay. A 10% SDS-PAGE gel was loaded with 20 μg of total protein. The antibodies, used for Western blot analysis, included rabbit polyclonal anti-SLC7A5 antibody (Cell signaling tech Inc., 1:1000), anti-GAPDH antibody (Kangchen Inc., 1:10000), and HRP-conjugated anti-rabbit secondary antibody (Kangchen Inc., 1:10000).

### Cell proliferation assay

Cell growth was determined by CCK-8 assay (Dojindo, Japan). Briefly, 5000 cells were seeded onto a 96-well plate in quadruplicate for each condition. CCK-8 reagent was added to each well in 10 μL after 4 hours after the cells were seeded, and then the cells were incubated for another 5 hours. Each sample was measured at 450nm and 630nm for its absorbance value at 0, 24, 48, 72h since the incubation.

### Cell apoptosis analysis

Cell apoptosis was determined by Annexin V Apoptosis Detection Kit APC (eBioscience Inc.). Approximately 2 × 10^5^ cells from each experimental group were washed with PBS and then suspended by annexin-binding buffer. Subsequently, 5μL annexin V and 5 μL diluted PI were added to each sample. Incubated for another 30 minutes, the cells were diluted by buffer and then analyzed with a flow cytometry.

### Cell cycle analysis

SKM-1 cells were exposed to various YSY01Aconcentrations for 48h, washed with twice cold PBS and harvested. The cell aggregate then was suspended in 70% ice-cold ethanol at −20°C overnight. The fixed cells were incubated with RNAase A for 30 min at 37°C, and stained with PI (propidiumidodide). The DNA content was detected using flow cytometry.

### Electron microscopy

Electron microscopy was conducted according to thestandard procedures. Approximately 0.25-mm-thick SKM-1 cell slices were postfixed briefly in 2 % osmium tetroxide in 0.1 M cacodylate buffer, dehydrated in graded ethanol and embedded in epoxy resin. Ultrathin 60–70 nm sections were cut using a Leica ultra-microtome, mounted on 200-meshcopper grids. Grids were examined and photographed using a transmission electron microscope (Philips CM200).

### Statistical analysis

All statistical analyses were performed using SPSS 15.0 software. Results for continuous variables were expressed as mean ± SD. The Kruskal-Wallis χ^2^-test was used to determine the significance of association between groups or proportions. Student's t-test was used to compare the means of results where appropriate. A value of P ≤ 0.05 was considered statistically significant.
